# Prognostic Impact of RAS and TP53 Mutation Profiles in Metastatic Colorectal Cancer

**DOI:** 10.3390/medicina62010136

**Published:** 2026-01-09

**Authors:** Mustafa Emre Duygulu, Elanur Karaman, Serdar Karakullukçu, Sevdegül Aydın Mungan

**Affiliations:** 1 Department of Medical Oncology, Erol Olçok Education and Research Hospital, Hitit University, 19000 Çorum, Türkiye; 2 Department of Medical Oncology, Faculty of Medicine, Karadeniz Technical University, 61080 Trabzon, Türkiye; drelanurkaraman@ktu.edu.tr; 3 Department of Public Health, Faculty of Medicine, Karadeniz Technical University, 61080 Trabzon, Türkiye; serdar.karakullukcu@ktu.edu.tr; 4 Department of Medical Pathology, Faculty of Medicine, Karadeniz Technical University, 61080 Trabzon, Türkiye; sevdegul@ktu.edu.tr

**Keywords:** metastatic colorectal carcinoma, metastasis, mutation, RAS, TP53

## Abstract

*Background and Objectives*: Our study aimed to investigate the effect of RAS and TP53 mutations, either alone or in combination, on survival in patients with metastatic colorectal carcinoma (mCRC). *Materials and Methods*: Patients diagnosed with mCRC and followed up at our center between January 2019 and November 2023, who underwent somatic mutation analysis via next-generation sequencing (NGS) testing, were retrospectively evaluated. A total of 155 patients were evaluated within the scope of the study. Patients were grouped as mutant type (m) or wild type (w) for RAS and TP53. Survival times between the groups were examined using the Kaplan–Meier method. Cox regression analysis was performed for factors with a prognostic effect on survival. *Results*: Among the patients, 35.4% exhibited an RASm/TP53w mutation profile, 30.9% had RASw/TP53w, 20% had RASw/TP53m, and 13.5% had RASm/TP53m. The lowest median progression-free survival (mPFS) and median overall survival (mOS) durations were observed in the RASm/TP53w group (7.3 months and 16.9 months, respectively). Median OS was significantly lower in the RASm/TP53w group compared to the RASw/TP53w group (16.9 months vs. 26.0 months, *p* = 0.003), whereas no significant difference was found between mPFS durations. No statistically significant difference was observed between the RASw/TP53m and RASm/TP53m groups and the RASw/TP53w group for mPFS and mOS. The RASm/TP53w mutation profile was identified as an independent prognostic factor for decreased OS in the multivariate Cox regression analysis. *Conclusions*: In mCRC cases with the RASm/TP53w mutation profile, the mOS was significantly lower. The RASm/TP53w mutation profile was identified as an independent prognostic factor for decreased OS. These findings are expected to contribute to the literature as real-world evidence regarding the prognostic value of different RAS and TP53 mutation combinations in mCRC.

## 1. Introduction

Colorectal cancer (CRC) is the third most common cancer worldwide and ranks second in cancer-related deaths [1]. Various molecular alterations have been identified in the development of CRC. These are often sporadic changes and are less frequently hereditary. Three main pathways play a role in CRC carcinogenesis: chromosomal instability, mismatch repair deficiency, and CpG island methylator phenotype (CIMP) pathways [2]. Knowledge of tumor mutations is critical in the management of metastatic colorectal cancer (mCRC) treatment; mutation analysis is required in every patient for personalized treatment approaches [3].

The RAS gene family includes the KRAS, NRAS, and HRAS genes, which play an important role in CRC carcinogenesis. RAS mutation is also associated with poor response to anti-EGFR targeted therapies and poor prognosis [4,5,6]. Despite differences between populations, the frequency of KRAS mutations in CRC cases is around 50%, while NRAS mutations are seen in around 4% of cases [7]. RAS mutations affect intracellular signaling pathways, and tumorigenesis is investigated through RAS-RAF-MEK-ERK pathway activation [8].

The TP53 gene is a tumor suppressor gene localized on the short arm of chromosome 17 and is the gene most frequently mutated in cancer cells. TP53 gene mutation leads to problems in DNA repair, apoptosis, checkpoint, and cellular aging processes. TP53 mutations have been reported in up to 60% of CRC cases [9,10]. KRAS and TP53 mutations are involved in colorectal carcinogenesis via the chromosomal instability pathway. RAS and TP53 mutations increase the angiogenic capacity, invasive capacity, and metastatic potential of tumor cells. They are also associated with treatment resistance and reduced survival times [11,12].

The prognosis in patients with CRC is influenced by the tumor stage, clinical, molecular, and histological characteristics. In patients with mCRC, the 5-year survival rate remains below 20% despite the use of chemotherapy and targeted agents. Tumor heterogeneity is thought to be the primary mechanism causing drug resistance [13,14].

Guidelines recommend that mismatch repair (MMR) status be assessed and mutation analysis of the KRAS, NRAS, and BRAF genes be performed in all patients before starting treatment, and that treatment be tailored according to these results in all patients diagnosed with mCRC [3]. With the widespread use of comprehensive genomic profiling, alterations in genes other than KRAS, NRAS, and BRAF mutations are also detected in mCRC cases. TP53 gene mutations are also among the most frequently mutated genes [15]. The number of studies investigating the effect of different combinations of RAS and TP53 mutations on mCRC prognosis is quite limited. Elucidating the clinical significance of these molecular alterations, either alone or in combination, may guide patient management. Our study aimed to investigate the effect of different combinations of RAS and TP53 mutations on survival times in mCRC patients as a retrospective single-center experience.

## 2. Materials and Methods

### 2.1. Study Design and Patients

Patients who were followed with a diagnosis of mCRC at our center between January 2019 and November 2023 were retrospectively evaluated. Data from a total of 155 patients were obtained from medical records and the electronic data system. Inclusion criteria were defined as being 18 years of age or older, having metastatic disease, having measurable distant organ metastases, having undergone Next Generation Sequencing (NGS) testing from tissue or liquid biopsy samples, and having accessible patient data. Exclusion criteria were defined as having an additional malignancy requiring active treatment, not having metastatic disease, and inaccessibility of patient data.

Biochemical analyses were performed using the BECKMAN COULTER AU 5821 analyzer (Beckman Coulter Diagnostics, Pasadena, CA, USA). The reference ranges were 0–3 U/mL for carcinoembryonic antigen (CEA) and 0–35 U/mL for carbohydrate antigen 19-9 (CA 19-9).

### 2.2. Statistical Analysis

Statistical analyses were performed using the SPSS version 26.0 statistical software package. Descriptive statistics of the evaluation results were presented as numbers and percentages for categorical variables, and as medians and minimum–maximum values for numerical variables. The normality of distribution within groups was assessed using the Kolmogorov–Smirnov test. Since numerical variables did not show normal distribution, the Kruskal–Wallis test was used for comparisons among three or more groups. The chi-square test was applied for comparisons of categorical variables between independent groups. Bonferroni correction was applied to reduce the probability of Type I error in multiple comparisons. Survival analysis was performed using the Kaplan–Meier method. Progression-free survival (PFS) was defined as the time from the diagnosis of metastatic disease to progression or death, and overall survival (OS) was defined as the time from the diagnosis of metastatic disease to death or the date of last follow-up. Cox regression analysis was conducted to identify factors independently associated with survival outcomes. A statistical alpha significance level of *p* < 0.05 was accepted.

### 2.3. Molecular Evaluation

In this study, the exonic regions and exon–intron junctions of 41 genes (ALK, APC, ATM, BCOR, BRAF, BRCA1, BRCA2, CDKN2A, CHEK2, DLX3 (DLL2), EGFR, ERBB2, ESR1, HRAS, IDH1, IDH2, KEAP1, KIT, KRAS, MET, MLH1, MSH2, MSH6, NFEL2 (NRF2), NRAS, NTRK1, NTRK2, NTRK3, PDGFRA, PIK3CA, PIK3R1, PMS2, POLE, PTEN, RAF1, ROS1, RPL22, STK11, TERT, TP53, and WNT1) were covered in solid large panel (CDHS-48998Z-2180) FFPE samples. NGS was performed on 147 patients using FFPE samples. NGS was performed on 8 patients using blood-based liquid biopsy.

The workflow covers sample extraction, library preparation, sequencing and bioinformatics steps. In the sample extraction step, DNA from FFPE samples is extracted via a QI-Aamp DNA FFPE Advanced Kit (Qiagen, Hilden, Germany). Then, a Qubit™ dsDNA HS, Thermo kit (Thermo Fisher Scientific, Waltham, MA, USA) with a Qubit™ 3 fluorometer was used to measure and optimize the DNA concentration. The CDHS-48998Z QIASeq Targeted DNA Human Custom Panel (Hilden, Germany) was used according to the manufacturer’s guidance. The extracted DNA is fragmented, and all the fragmented DNA is barcoded with unique molecular indices to track the original DNA molecules and provide high-sensitivity detection. Then, the targeted genes are amplified with single primer extension technology, and a bead clean-up step is performed to discard unwanted fragments. The concentration optimization of the libraries was performed with a Qiaseq Quant Assay Kit (Qiagen, Hilden, Germany), and all the libraries were diluted to 4 nM. Libraries with different sample indexes are combined in equimolar amounts in the final pool. The final pool is subsequently sequenced in the AVITI System, ELEMENT, according to the manufacturer’s instructions. The secondary analysis of fastq files is performed on the Qiagen CLC Genomic Workbench with a panel-specific pipeline. The vcf files are clinically interpreted via Qiagen Clinical Insight-Interpret. Microsatellite instability (MSI) was examined at 9 loci [BAT40(T)37, MONO-27(T)27, BAT26(A)27, NR24(T)23, BAT25(T)25, NR22(T)21, HSP110-T17(T)17, NR21(A)21 and BAT34C4(A)18].

Loss of expression detected in at least one of the MLH1, MSH2, MSH6, and PMS2 proteins via immunohistochemical staining was defined as deficient mismatch repair (dMMR).

## 3. Results

### 3.1. Study Population

A total of 155 patients diagnosed with mCRC were evaluated in the study. The median (min–max) age of all patients was 60 (23–85). There was no significant difference in age between mutation groups (*p* = 0.199). The primary tumor was located in the colon in 89 patients (57.4%), in the rectum in 55 patients (35.5%), and in the rectosigmoid in 11 patients (7.1%). RASw/TP53m tumors were mostly localized in the rectum (45.2%), while RASm/TP53m tumors were mostly (76.2%) localized in the colon (*p* = 0.048). The primary tumor was located on the left side in 81.3% of patients and on the right side in 18.7% (*p* = 0.083). Liver metastases were present in 105 cases (67.7%), lung metastases in 58 cases (37.4%), nonregional lymph node metastases in 46 cases (29.7%), and ascites/peritoneal metastases in 24 cases (15.5%). No significant difference was observed between mutation groups in terms of metastatic sites (*p* = 0.415 for lung, *p* = 0.254 for liver, *p* = 0.278 for ascites/peritoneum, *p* = 0.994 for nonregional lymph node). Curative metastasectomy was performed in 13 patients (8.4%), and there was no difference between the groups (*p* = 0.181). NGS was performed on the primary tumor in 137 (88.3%) patients, on metastatic tissue in 10 (6.4%) patients, and on blood liquid biopsy in 8 (5.1%) patients. The clinical and demographic characteristics of all cases are shown in Table 1.

**Figure 1:**
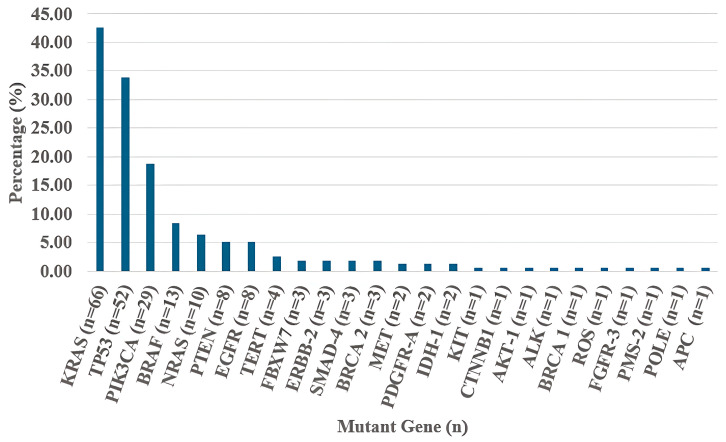
Frequency and distribution of somatic mutations in the study cohort.

KRAS mutations were detected in 66 patients (42.5%), TP53 mutations in 52 patients (39.9%), and NRAS mutations in 10 patients (6.4%). Patients were divided into 4 groups based on mutation analysis results. RAS mutant and TP53 wild (RASm/TP53w) (*n* = 55, 35.4%), RAS wild and TP53 wild (RASw/TP53w) (*n* = 48, 30.9%), RAS wild and TP53 mutant (RASw/TP53m) (*n* = 31, 20%), RAS mutant and TP53 mutant (RASm/TP53m) (*n* = 21, 13.5%). The number of deficient mismatch repair/high microsatellite instability (dMMR/MSI-H) patients was 10 (6.5%). There was no significant difference in mutation profiles for dMMR/MSI-H status (*p* = 0.083). The distribution of all somatic mutations is shown in Figure 1.

### 3.2. Survival Outcomes

Median PFS (mPFS) in the entire population was 9.4 months (95% CI 8.0–10.7), and mPFS in those with RASw/TP53w was 10.2 months (95% CI 9.0–11.3), and in those with RASm/TP53w it was 7.3 months (95% CI 6.0–8.6), 10.6 months in those with RASw/TP53m (95% CI 7.3–13.8), and 10.0 months in those with RASm/TP53m (95% CI 8.3–11.6) (*p* = 0.296).

The median overall survival (mOS) in the entire population was 19.9 months (95% CI 14.6–25.2), 26.0 months (95% CI 16.6–35.3) in those with RASw/TP53w, 16.9 months (95% CI 15.2–18.7) in those with RASm/TP53w, 24.8 months in those with RASw/TP53m (95% CI 19.2–30.3), and 18.5 months in those with RASm/TP53m (95% CI 9.1–27.9) (*p* = 0.026).

**Figure 2:**
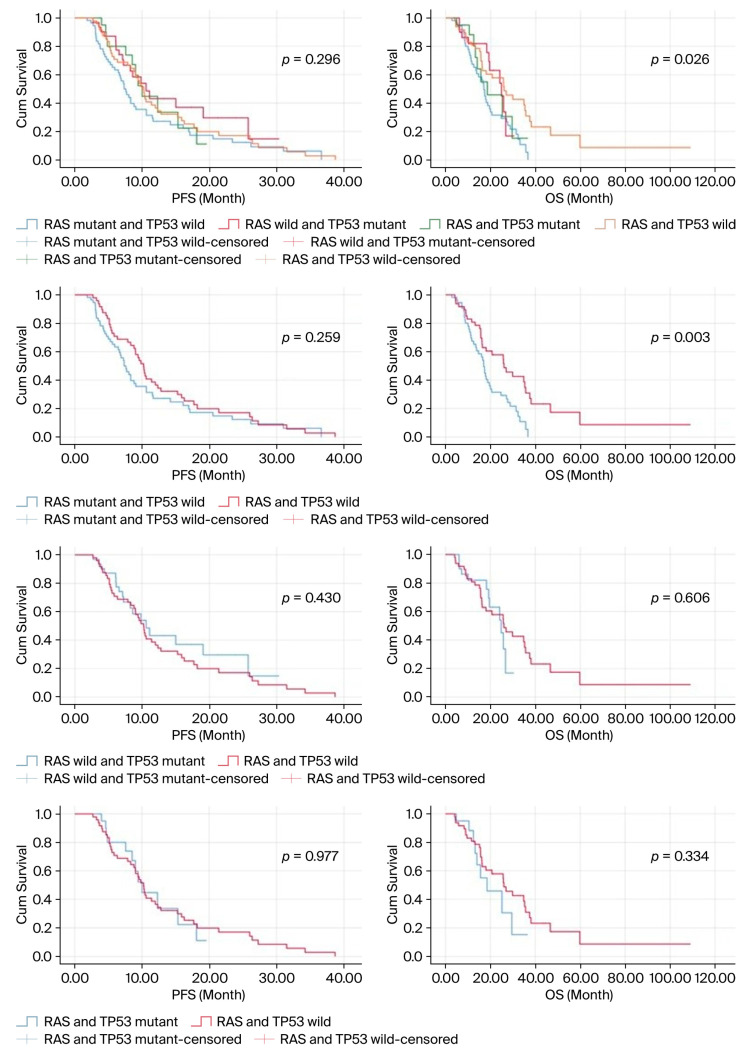
Kaplan–Meier curves for survival according to patients’ mutation profiles.

No difference in mPFS was observed between those with the RASm/TP53w mutation profile and those with the RASw/TP53w mutation profile (*p* = 0.259), but a significant decrease was observed in mOS durations (*p* = 0.003). No significant difference was detected in mPFS and mOS between patients with the RASw/TP53m mutation profile and the RASw/TP53w group (*p* = 0.430 and *p* = 0.606, respectively). Although the mPFS and mOS values were numerically lower in the RASm/TP53m group compared to the RASw/TP53w group, this did not reach statistical significance (*p* = 0.977 and *p* = 0.334, respectively). Survival Kaplan–Meier curves based on the mutation analysis results of the cases are shown in Figure 2.

Elevated CA 19-9 levels were identified as an independent prognostic factor for reduced PFS (Hazard ratio (HR) 1.64 (1.13–2.37), *p* = 0.008) in multivariate Cox regression analysis (Table 2). Additionally, the RASm/TP53w mutation profile was identified as an independent prognostic factor for reduced OS (Hazard ratio (HR) 1.89 (1.13–3.16), *p* = 0.015) (Table 3).

## 4. Discussion

In our study, the prognostic significance of the presence of RAS and TP53 mutations individually or in combination was evaluated in metastatic colorectal carcinoma. Patients with the RASm/TP53w mutation profile had significantly shorter median overall survival (mOS) durations compared with the RASw/TP53w group. In the RASm/TP53m and RASw/TP53m groups, no significant differences were observed in median overall survival (mOS) or median progression-free survival (mPFS) when compared with the RASw/TP53w group. The RASm/TP53w mutation profile was identified as an independent prognostic indicator for decreased OS.

It has been reported that the process leading from normal epithelial cells to cancer begins with APC gene inactivation, KRAS activation occurs in the early stages, and TP53 mutations are observed in the late stages of colorectal carcinogenesis [16]. Proteins encoded by the KRAS, NRAS, and HRAS genes of the RAS oncogene family bind guanosine triphosphate (GTP) and become activated within the cell, subsequently activating the RAF-MEK-ERK pathway and the PI3K-AKT-mTOR signaling pathways. Cell proliferation, differentiation, and apoptosis are regulated through these intracellular signaling pathways [17]. Studies have shown that KRAS and NRAS mutations are associated with reduced response to anti-EGFR therapies [18,19].

TP53, an important tumor suppressor gene, plays an antiproliferative role against various cellular stress factors through the proteins it encodes. TP53 mutation is the most common mutation observed in cancer cells and has been reported to be associated with poor prognosis in different types of cancer [20,21,22,23]. In the literature, the coexistence of KRAS and P53 mutations in patients with locally advanced rectal adenocarcinoma has been reported to be associated with the failure to achieve a pathological complete response (pCR) after neoadjuvant chemoradiotherapy [24].

In a study by Chun et al. involving 401 CRC patients who underwent liver metastasectomy, the coexistence of RAS and TP53 mutations was reported to be an independent risk factor for decreased OS, whereas the presence of these mutations individually was not found to have prognostic significance in multivariate analysis [25]. Similarly, we observed that isolated TP53 mutation did not affect survival outcomes. In contrast, RASm/TP53w mutation profile was associated with decreased OS in multivariate analysis. Unlike the referenced study, the number of patients who underwent curative metastasectomy in our cohort was limited, and most of our patients had de novo metastatic disease with measurable distant metastases. Additionally, the number of patients with the RASm/TP53m profile in our population was low. These differences limit direct comparison between the two studies.

Wang et al. investigated the clinical significance of KRAS mutation and P53 expression in locally advanced operable BRAF wild-type CRC patients. P53 expression was assessed by immunohistochemistry, while KRAS mutation was evaluated by PCR. It was reported that OS was significantly lower in the group with KRAS mutation and high P53 expression. However, isolated KRAS mutation or P53 expression was shown not to affect survival [26]. NGS, a more comprehensive method capable of evaluating multiple genes was performed in our study. Although survival durations were lower in the RASm/TP53m subgroup, the results did not reach statistical significance. This was thought to be primarily attributable to differences in disease stage between study populations, as tumor behavior may vary between early-stage and metastatic disease. Additionally, the methods used for mutation detection differed across studies.

In early-stage CRC patients, those with dMMR/MSI-H have a better prognosis compared with proficient mismatch repair/microsatellite stable (pMMR/MSS) patients [27,28]. A pooled analysis of four studies involving 3063 patients showed that dMMR was associated with shorter survival in patients with metastatic colon cancer, and it has been suggested that this may be related to the higher number of BRAF mutations in dMMR patients [29]. The absence of dMMR/MSI-H patients in the RASm/TP53m profile in our study may have led to better survival outcomes in this subgroup. At the same time, the absence of patients treated with immunotherapy among dMMR/MSI-H patients may have contributed to the poorer survival outcomes in other groups.

The PI3K/AKT/mTOR signaling pathway also plays an important role in CRC. Mutations in the PIK3CA gene are reported in approximately one-third of patients [30]. Resistance to chemotherapy and anti-EGFR therapies is known to occur in those with this mutation [31]. In our study, approximately one-third of the RASm/TP53m group had a PIK3CA mutation. Survival durations were numerically shorter, but no statistical difference was obtained. Due to the relatively small number of patients in the mutation subgroups, it was thought that the effect of the PIK3CA mutation on survival times could not be clearly demonstrated.

It has been reported that KRAS mutations are more common in the right-sided colon cancer, while TP53 mutations are more common in the left-sided colon cancer [32]. Our study did not reveal a significant relationship between mutation subgroups and tumor side. It may be related to the characteristics of this study population and the limited number of patients in the mutation subgroups. Additionally, tumors were located on the left side in more than 80% of patients, which was thought to potentially interfere with measuring the difference between tumor side and mutation profile.

The prognosis in CRC patients is influenced by many factors. These include tumor location, genetic mutations, disease stage, access to targeted therapies and immunotherapy, local treatment applications, and population-related variables. The effects of mutations on biological behavior may differ between early-stage and metastatic disease, and the emergence of new mutations under treatment may also contribute to therapeutic resistance. It is known that the RAS mutation profile in patients with metastatic colorectal cancer may change over time under systemic treatment [33]. In our study, NGS testing was performed before initiating first-line treatment for metastatic disease. Approximately 70% of patients had de novo metastatic disease, and NGS analysis was performed using primary tumor tissue in 88% of patients.

The main limitations of our study are its retrospective design and single-center experience. Furthermore, the limited number of patients in the general population and in the mutation subgroups precluded the generalization of statistical analyses, preventing clear comparisons with literature data and necessitating cautious interpretation. Additionally, the effect of mutation profiles other than RAS and TP53 on survival could not be evaluated due to the limited number of patients in the subgroups.

## 5. Conclusions

In this study, the clinical significance of different combinations of RAS and TP53 mutations—identified through comprehensive genomic profiling, which is widely used today—was evaluated in patients with metastatic colorectal carcinoma. The RASm/TP53w mutation profile was found to be associated with reduced overall survival and poor prognosis. Although overall survival durations were lower in patients with RASm/TP53m and RASw/TP53m compared with those with RASw/TP53w, this decrease could not be demonstrated to have an impact on prognosis.

Given the limited data available on the clinical impact of different alterations in RAS and TP53 mutations in metastatic colorectal carcinoma, this study is considered to contribute to the literature as a real-life experience. Validation of these findings through large, multicenter, prospective studies will contribute to the clinical interpretation of these mutations in patients with metastatic colorectal carcinoma.

## Figures and Tables

**Table 1:** Clinical and pathological characteristics of the patients.

Variable		Total *n*-%	RASw/TP53w (1) (*n* = 48, 30.9%)	RASm/TP53w (2) (*n* = 55, 35.4%)	RASw/TP53m (3) (*n* = 31, 20%)	RASm/TP53m (4) (*n* = 21, 13.5%)	*p*	*p* (1–4) *	*p* (2–4) *	*p* (3–4) *
**Age**	Med-min max	60 (23–85)	58 (37–85)	59 (23–82)	60 (36–80)	65 (34–81)	0.199 ^1^	0.156 ^1^	0.630 ^1^	0.582 ^1^
	65<	100 (64.5)	34 (70.8)	35 (63.6)	21 (67.7)	10 (47.6)	0.306 ^2^	0.390 ^2^	1.000 ^2^	0.882 ^2^
	65≥	55 (35.5)	14 (29.2)	20 (36.4)	10 (32.3)	11 (52.4)				
**Gender**	Female	65 (41.9)	17 (35.4)	28 (50.9)	8 (25.8)	12 (57.1)	0.047 ^2^	0.558 ^2^	1.000 ^2^	0.138 ^2^
	Male	90 (58.1)	31 (64.6)	27 (49.1)	23 (74.2)	9 (42.9)				
**Primary tumor**	Rectum	55 (35.5)	16 (33.3)	20 (36.4)	14 (45.2)	5 (23.8)	0.038 ^2^	1.000 ^2^	1.000 ^2^	0.048 ^2^
	Rectosigmoid	11 (7.1)	3 (6.3)	2 (3.6)	6 (19.4)	0 (0.0)				
	Colon	89 (57.4)	29 (60.4)	33 (60.0)	11 (35.5)	16 (76.2)				
**Tumor side**	Right	29 (18.7)	10 (20.8)	14 (25.5)	1 (3.2)	4 (19.0)	0.083 ^2^	1.000 ^2^	1.000 ^2^	0.870 ^2^
	Left	126 (81.3)	38 (79.2)	41 (74.5)	30 (96.8)	17 (81.0)				
**Metastasis status**	De novo	108 (69.7)	32 (66.7)	37 (67.3)	24 (77.4)	15 (71.4)	0.737 ^2^	1.000 ^2^	1.000 ^2^	1.000 ^2^
	Recurrent	47 (30.3)	16 (33.3)	18 (32.7)	7 (22.6)	6 (28.6)				
**Metastasectomy**	Present	13 (8.4)	4 (8.3)	7 (12.7)	0 (0.0)	2 (9.5)	0.181 ^2^	1.000 ^2^	1.000 ^2^	0.948 ^2^
	Absent	142 (91.6)	44 (91.7)	48 (87.3)	31 (100.0)	19 (90.5)				
**Number of metastatic sites**	<3	141 (91.0)	45 (94.0)	50 (90.9)	29 (93.5)	17 (81.0)	0.390 ^2^	1.000 ^2^	1.000 ^2^	1.000 ^2^
	≥3	14 (9.0)	3 (6.0)	5 (9.1)	2 (6.5)	4 (19.0)				
**Metastatic site**	Lung	58 (37.4)	15 (31.3)	21 (38.2)	11 (35.5)	11 (52.4)	0.415 ^2^	0.576 ^2^	1.000 ^2^	1.000 ^2^
	Liver	105 (67.7)	35 (72.9)	40 (72.7)	19 (61.3)	11 (52.4)	0.254 ^2^	0.576 ^2^	0.546 ^2^	1.000 ^2^
	Ascites/Peritoneum	24 (15.5)	5 (10.4)	7 (12.7)	7 (22.6)	5 (23.8)	0.278 ^2^	0.954 ^2^	1.000 ^2^	1.000 ^2^
	Nonregional lymph nodes	46 (29.7)	15 (31.3)	16 (29.1)	9 (29.0)	6 (28.6)	0.994 ^2^	1.000 ^2^	1.000 ^2^	1.000 ^2^
**dMMR/MSI-H**	dMMR/MSI-H	10 (6.5)	6 (12.5)	2 (3.6)	2 (6.5)	0 (0.0)	0.083 ^2^	0.390 ^2^	1.000 ^2^	1.000 ^2^
	pMMR/MSI-L	134 (86.5)	37 (77.1)	47 (85.5)	29 (93.5)	21 (100.0)				
	Not evaluated	11 (7.1)	5 (10.4)	6 (10.9)	0 (0.0)	0 (0.0)				
**CEA**	High	121 (78.1)	35 (72.9)	43 (78.2)	25 (80.6)	18 (85.7)	0.664 ^2^	1.000 ^2^	1.000 ^2^	1.000 ^2^
	Normal	34 (21.9)	13 (27.1)	12 (21.8)	6 (19.4)	3 (14.3)				
**CA19-9**	High	80 (51.6)	20 (41.7)	32 (58.2)	14 (45.2)	14 (66.7)	0.153 ^2^	0.336 ^2^	1.000 ^2^	0.762 ^2^
	Normal	75 (48.4)	28 (58.3)	23 (41.8)	17 (54.8)	7 (33.3)				
**PIK3CA mutation**	Present	29 (18.7)	6 (12.5)	14 (25.5)	2 (6.5)	7 (33.3)	0.031 ^2^	0.318 ^2^	1.000 ^2^	0.132 ^2^
	Absent	126 (81.3)	42 (87.5)	41 (74.5)	29 (93.5)	14 (66.7)				
**BRAF mutation**	Present	13 (8.4)	8 (16.7)	4 (7.3)	0 (0.0)	1 (4.8)	0.058 ^2^	1.000 ^2^	1.000 ^2^	1.000 ^2^
	Absent	142 (91.6)	40 (83.3)	51 (92.7)	31 (100.0)	20 (95.2)				
**Other mutations**	Present	25 (16.1)	8 (16.7)	13 (23.6)	2 (6.5)	2 (9.5)	0.163 ^2^	1.000 ^2^	1.000 ^2^	1.000 ^2^
	Absent	130 (83.9)	40 (83.3)	42 (76.4)	29 (93.5)	19 (90.5)				
**First line therapy**	ChT+Anti-EGFR	53 (34.2)	28 (58.3)	1 (1.8)	24 (77.4)	0 (0.0)	<0.001 ^2^	<0.006 ^2^	1.000 ^2^	<0.006 ^2^
	ChT+AntiVEGF	64 (41.3)	7 (14.6)	39 (70.9)	1 (3.2)	17 (81.0)				
	Other regimes	38 (24.5)	13 (27.1)	15 (27.3)	6 (19.4)	4 (19.0)				

^1^ Kruskal–Wallis Test; ^2^ Chi-squared test; * Bonferroni correction was applied; MSI-H: High microsatellite instability; MSI-L: Low microsatellite instability; dMMR: Deficient mismatch repair; pMMR: Proficient mismatch repair; CEA: Carcinoembryonic antigen; CA 19-9: Carbohydrate antigen 19-9; CEA: Carcinoembryonic antigen; ChT: Chemotherapy; EGFR: Epidermal growth factor receptor; VEGF: Vascular endothelial growth factor; w: wild; m: mutant.

**Table 2:** Cox Regression Analysis of factors associated with progression-free survival.

Variables		Univariate Analysis HR (95% CI)	*p*	Multivariate Analysis HR (95% CI)	*p*
**Age**	65 < ref.	1			
	65≥	0.87 (0.59–1.27)	0.485		
**Gender**	Female ref.	1			
	Male	0.86 (0.59–1.23)	0.416		
**Tumor side**	Right ref.	1		1	
	Left	0.59 (0.37–0.94)	0.028	0.62 (0.39–1.00)	0.051
**Metastasis status**	De novo ref.	1			
	Recurrent	1.31 (0.88–1.94)	0.174		
**MSI/MMR**	dMMR/MSI-H ref.	1			
	pMMR/MSI-L	1.01 (0.49–2.09)	0.960		
**CEA**	Normal ref.	1			
	High	1.14 (0.72–1.81)	0.569		
**CA 19-9**	Normal ref.	1		1	
	High	1.68 (1.16–2.41)	0.005	1.64 (1.13–2.37)	0.008
**Metastatic site**	<3 ref.	1		1	
	3≥	1.69 (0.94–3.03)	0.077	1.28 (0.70–2.36)	0.414
**PIK3CA mutation**	Absent ref.	1			
	Present	0.94 (0.60–1.48)	0.799		
**BRAF mutation**	Absent ref.	1			
	Present	0.98 (0.53–1.84)	0.970		
**Other mutations**	Absent ref.	1			
	Present	1.33 (0.83–2.11)	0.229		
**Mutation status**	RAS and TP53 wild ref.	1			
	RAS mutant and TP53 wild	1.28 (0.84–1.94)	0.237		
	RAS wild and TP53 mutant	0.78 (0.45–1.37)	0.402		
	RAS and TP53 mutant	0.91 (0.48–1.75)	0.799		

MSI-H: High microsatellite instability; MSI-L: Low microsatellite instability; dMMR: Deficient mismatch repair; pMMR: Proficient mismatch repair; CEA: Carcinoembryonic antigen; CA 19-9: Carbohydrate antigen 19-9; ref.: Reference.

**Table 3:** Cox Regression Analysis of factors associated with overall survival.

Variables		Univariate Analysis HR (95% CI)	*p*	Multivariate Analysis HR (95% CI)	*p*
**Age**	65 < ref.	1			
	65≥	0.92 (0.60–1.41)	0.704		
**Gender**	Female ref.	1			
	Male	0.85 (0.56–1.28)	0.452		
**Tumor side**	Right ref.	1			
	Left	0.75 (0.46–1.21)	0.241		
**Metastasis status**	De novo ref.	1			
	Recurrent	1.02 (0.65–1.59)	0.913		
**MSI/MMR**	dMMR/MSI-H ref.	1			
	pMMR/MSI-L	1.63 (0.69–3.81)	0.260		
**CEA**	Normal ref.	1			
	High	1.51 (0.85–2.69)	0.152		
**CA 19-9**	Normal ref.	1		1	
	High	1.53 (1.01–2.33)	0.042	1.29 (0.83–2.01)	0.249
**Metastatic site**	<3 ref.	1		1	
	3≥	1.76 (0.93–3.33)	0.081	1.42 (0.73–2.75)	0.290
**PIK3CA mutation**	Absent ref.	1			
	Present	0.65 (0.37–1.14)	0.140		
**BRAF mutation**	Absent ref.	1			
	Present	1.10 (0.55–2.19)	0.786		
**Other mutations**	Absent ref.	1			
	Present	1.15 (0.68–1.97)	0.586		
**Mutation status**	RAS and TP53 wild ref.	1		1	
	RAS mutant and TP53 wild	2.09 (1.27–3.44)	0.003	1.89 (1.13–3.16)	0.015
	RAS wild and TP53 mutant	1.31 (0.65–2.63)	0.448	1.29 (0.64–2.61)	0.464
	RAS and TP53 mutant	1.53 (0.71–3.30)	0.272	1.39 (0.64–3.02)	0.402

MSI-H: High microsatellite instability; MSI-L: Low microsatellite instability; dMMR: Deficient mismatch repair; pMMR: Proficient mismatch repair; CEA: Carcinoembryonic antigen; CA 19-9: Carbohydrate antigen 19-9; ref.: Reference.

## Data Availability

The datasets presented in this article are not publicly available due to the inclusion of genetic information.

## Abbreviations

The following abbreviations are used in this manuscript:

CRC	Colorectal carcinoma
RASw	RAS wild
RASm	RAS mutant
TP53w	TP53 wild
TP53m	TP53 mutant
CIMP	CpG island methylator phenotype
PFS	Progression-free survival
OS	Overall survival
dMMR	Deficient mismatch repair
pMMR	Proficient mismatch repair
MSI-H	High microsatellite instability
MSI-L	Low microsatellite instability
GTP	Guanosine triphosphate
PCR	Pathological complete response
CA 19-9	Carbohydrate antigen 19-9
ChT	Chemotherapy
EGFR	Epidermal growth factor receptor
VEGF	Vascular endothelial growth factor

## References

[1] Bray F., Laversanne M., Sung H., Ferlay J., Siegel R.L., Soerjomataram I., Jemal A. (2024). Global cancer statistics 2022: GLOBOCAN estimates of incidence and mortality worldwide for 36 cancers in 185 countries. CA Cancer J. Clin..

[2] Nguyen L.H., Goel A., Chung D.C. (2020). Pathways of Colorectal Carcinogenesis. Gastroenterology.

[3] Cervantes A., Adam R., Roselló S., Arnold D., Normanno N., Taïeb J., Seligmann J., De Baere T., Osterlund P., Yoshino T. (2023). Metastatic colorectal cancer: ESMO Clinical Practice Guideline for diagnosis, treatment and follow-up. Ann. Oncol..

[4] Modest D.P., Ricard I., Heinemann V., Hegewisch-Becker S., Schmiegel W., Porschen R., Stintzing S., Graeven U., Arnold D., von Weikersthal L.F. (2016). Outcome according to KRAS-, NRAS- and BRAF-mutation as well as KRAS mutation variants: Pooled analysis of five randomized trials in metastatic colorectal cancer by the AIO colorectal cancer study group. Ann. Oncol..

[5] Cercek A., Braghiroli M.I., Chou J.F., Hechtman J.F., Kemeny N., Saltz L., Capanu M., Yaeger R. (2017). Clinical Features and Outcomes of Patients with Colorectal Cancers Harboring NRAS Mutations. Clin. Cancer Res..

[6] Saeed O., Lopez-Beltran A., Fisher K.W., Scarpelli M., Montironi R., Cimadamore A., Massari F., Santoni M., Cheng L. (2019). RAS genes in colorectal carcinoma: Pathogenesis, testing guidelines and treatment implications. J. Clin. Pathol..

[7] Prior I.A., Hood F.E., Hartley J.L. (2020). The Frequency of Ras Mutations in Cancer. Cancer Res..

[8] Song Y., Bi Z., Liu Y., Qin F., Wei Y., Wei X. (2022). Targeting RAS-RAF-MEK-ERK signaling pathway in human cancer: Current status in clinical trials. Genes Dis..

[9] Manirakiza F., Yamada H., Iwashita Y., Ishino K., Ishikawa R., Kovacs Z., Osvath E., Nzitakera A., Gurzu S., Sugimura H. (2023). TP53 mutations in Romanian patients with colorectal cancer. Genes Environ..

[10] Hernández Borrero L.J., El-Deiry W.S. (2021). Tumor suppressor p53: Biology, signaling pathways, and therapeutic targeting. Biochim. Biophys. Acta Rev. Cancer.

[11] Rachmawati M., Yulianti H., Hernowo B.S., Suryanti S., Wijaya I., Rahadiani N., Heriyanto D.S., Irianiwati I. (2019). The Correlation of KRAS Gene Expression and P53 Immunoexpression in Colorectal Adenocarcinoma. Open Access Maced. J. Med. Sci..

[12] Zhu G., Pei L., Xia H., Tang Q., Bi F. (2021). Role of oncogenic KRAS in the prognosis, diagnosis and treatment of colorectal cancer. Mol. Cancer.

[13] Rumpold H., Niedersüß-Beke D., Heiler C., Falch D., Wundsam H.V., Metz-Gercek S., Piringer G., Thaler J. (2020). Prediction of mortality in metastatic colorectal cancer in a real-life population: A multicenter explorative analysis. BMC Cancer.

[14] Leiphrakpam P.D., Rajappa S.J., Krishnan M., Batra R., Murthy S.S., Are C. (2023). Colorectal cancer: Review of signaling pathways and associated therapeutic strategies. J. Surg. Oncol..

[15] Su Z., El Hage M., Linnebacher M. (2024). Mutation patterns in colorectal cancer and their relationship with prognosis. Heliyon.

[16] Pierantoni C., Cosentino L., Ricciardiello L. (2024). Molecular Pathways of Colorectal Cancer Development: Mechanisms of Action and Evolution of Main Systemic Therapy Compunds. Dig. Dis..

[17] Cuesta C., Arévalo-Alameda C., Castellano E. (2021). The Importance of Being PI3K in the RAS Signaling Network. Genes.

[18] Douillard J.Y., Oliner K.S., Siena S., Tabernero J., Burkes R., Barugel M., Humblet Y., Bodoky G., Cunningham D., Jassem J. (2013). Panitumumab-FOLFOX4 treatment and RAS mutations in colorectal cancer. N. Engl. J. Med..

[19] Takeda M., Yoshida S., Inoue T., Sekido Y., Hata T., Hamabe A., Ogino T., Miyoshi N., Uemura M., Yamamoto H. (2025). The Role of KRAS Mutations in Colorectal Cancer: Biological Insights, Clinical Implications, and Future Therapeutic Perspectives. Cancers.

[20] Vogelstein B., Lane D., Levine A.J. (2000). Surfing the p53 network. Nature.

[21] Hainaut P., Hollstein M. (2000). p53 and human cancer: The first ten thousand mutations. Adv. Cancer Res..

[22] Olivier M., Hollstein M., Hainaut P. (2010). TP53 mutations in human cancers: Origins, consequences, and clinical use. Cold Spring Harb. Perspect. Biol..

[23] Chen X., Zhang T., Su W., Dou Z., Zhao D., Jin X., Lei H., Wang J., Xie X., Cheng B. (2022). Mutant p53 in cancer: From molecular mechanism to therapeutic modulation. Cell Death Dis..

[24] Garcia-Aguilar J., Chen Z., Smith D.D., Li W., Madoff R.D., Cataldo P., Marcet J., Pastor C. (2011). Identification of a biomarker profile associated with resistance to neoadjuvant chemoradiation therapy in rectal cancer. Ann. Surg..

[25] Chun Y.S., Passot G., Yamashita S., Nusrat M., Katsonis P., Loree J.M., Conrad C., Tzeng C.D., Xiao L., Aloia T.A. (2019). Deleterious Effect of RAS and Evolutionary High-risk TP53 Double Mutation in Colorectal Liver Metastases. Ann. Surg..

[26] Wang L., Lin S., Yang C., Cai S., Li W. (2022). Effect of KRAS mutations and p53 expression on the postoperative prognosis of patients with colorectal cancer. Mol. Genet. Genom. Med..

[27] French A.J., Sargent D.J., Burgart L.J., Foster N.R., Kabat B.F., Goldberg R., Shepherd L., Windschitl H.E., Thibodeau S.N. (2008). Prognostic significance of defective mismatch repair and BRAF V600E in patients with colon cancer. Clin. Cancer Res..

[28] Dienstmann R., Mason M.J., Sinicrope F.A., Phipps A.I., Tejpar S., Nesbakken A., Danielsen S.A., Sveen A., Buchanan D.D., Clendenning M. (2017). Prediction of overall survival in stage II and III colon cancer beyond TNM system: A retrospective, pooled biomarker study. Ann. Oncol..

[29] Venderbosch S., Nagtegaal I.D., Maughan T.S., Smith C.G., Cheadle J.P., Fisher D., Kaplan R., Quirke P., Seymour M.T., Richman S.D. (2014). Mismatch repair status and BRAF mutation status in metastatic colorectal cancer patients: A pooled analysis of the CAIRO, CAIRO2, COIN, and FOCUS studies. Clin. Cancer Res..

[30] Leiphrakpam P.D., Chowdhury S., Wang J., Black J.D., Are C. (2021). The role and therapeutic implications of PI3K signaling pathway in cancer. J. Surg. Oncol..

[31] Wu S., Gan Y., Wang X., Liu J., Li M., Tang Y. (2013). PIK3CA mutation is associated with poor survival among patients with metastatic colorectal cancer following anti-EGFR monoclonal antibody therapy: A meta-analysis. J. Cancer Res. Clin. Oncol..

[32] Puccini A., Marshall J.L., Salem M.E. (2018). Molecular variances between right-and left-sided colon cancers. Curr. Color. Cancer Rep..

[33] Stintzing S., Klein-Scory S., Fischer von Weikersthal L., Fuchs M., Kaiser F., Heinrich K., Modest D.P., Hofheinz R.D., Decker T., Gerger A. (2025). Baseline Liquid Biopsy in Relation to Tissue-Based Parameters in Metastatic Colorectal Cancer: Results From the Randomized FIRE-4 (AIO-KRK-0114) Study. J. Clin. Oncol..

